# Enhancing the Immunogenicity of RBD Protein Variants through Amino Acid E484 Mutation in SARS-CoV-2

**DOI:** 10.3390/v14092020

**Published:** 2022-09-13

**Authors:** Zhikai Zhang, Xuan Wan, Xinyue Li, Shaoxi Cai, Chengsong Wan

**Affiliations:** 1 BSL-3 Laboratory (Guangdong), Guangdong Provincial Key Laboratory of Tropical Disease Research, School of Public Health, Southern Medical University, Guangzhou 510515, China; 2 Chronic Airways Diseases Laboratory, Department of Respiratory and Critical Care Medicine, Nanfang Hospital, Southern Medical University, Guangzhou 510515, China

**Keywords:** COVID-19, SARS-CoV-2, RBD, immunogenicity, mutant

## Abstract

In the context of the COVID-19 pandemic, conducting antibody testing and vaccination is critical. In particular, the continued evolution of SARS-CoV-2 raises concerns about the effectiveness of vaccines currently in use and the activity of neutralizing antibodies. Here, we used the *Escherichia coli* expression system to obtain nine different SARS-CoV-2 RBD protein variants, including six single-point mutants, one double-point mutant, and two three-point mutants. Western blotting results show that nine mutants of the RBD protein had strong antigenic activity in vitro. The immunogenicity of all RBD proteins was detected in mice to screen for protein mutants with high immunogenicity. The results show that the mutants E484K, E484Q, K417T-E484K-N501Y, and K417N-E484K-N501Y, especially the former two, had better immunogenicity than the wild type. This suggests that site E484 has a significant impact on the function of the RBD protein. Our results demonstrate that recombinant RBD protein expressed in *E. coli* can be an effective tool for the development of antibody detection methods and vaccines.

## 1. Introduction

The beta-coronavirus SARS-CoV-2 has become the seventh discrete coronavirus species that is capable of causing human disease [1]. SARS-CoV-2 is easily transmitted and highly pathogenic [2,3]. There are now outbreaks in more than 216 countries, areas, and territories around the world. As of 27 July 2022, the total number of confirmed cases has exceeded 570 million, with more than 6.3 million deaths, and these numbers are increasing every day. Vaccination is a highly effective strategy to prevent and stop the spread of SARS-CoV-2 in light of its high pathogenicity and transmissible nature. Well-protected vaccines can significantly reduce the incidence and transmission of the virus and are of great significance to the prevention and treatment of COVID-19. Many different SARS-CoV-2 vaccines are being developed around the world. According to the technology, the current vaccines are divided into three main categories: novel coronavirus-inactivated vaccines, subunit recombinant protein vaccines, and nucleic acid vaccines [4,5].

Studies have shown that SARS-CoV-2 spike (S) protein can serve as a suitable antigen with strong immunogenicity that can effectively stimulate the host immune system to induce the production of neutralizing antibodies [5,6,7]. The receptor-binding domain (RBD) in the SARS-CoV-2 S protein mediates viral cell fusion to induce host infection through site-directed binding to the receptor protein angiotensin-converting enzyme 2 (ACE2) [8,9]. Therefore, the S protein has become a priority target for the design of recombinant subunit vaccines [5,10,11]. SARS-CoV-2 was first detected in 2019 [12]. As the virus has spread globally, new variants of the virus have emerged in the past two years, some of which have significantly increased their infectivity, transmissibility, and immune escape potential compared to wild viruses [13,14,15]. In multiple variants of SARS-CoV-2 that have been reported so far, the S protein has been mutated, especially at some sites in the RBD, resulting in an increase in the binding affinity between the S protein and the receptor protein ACE2 [16,17]. For example, the RBD mutation N501Y appears in lineages B.1.1.7 (Alpha), B.1.351 (Beta), P.1 (Gamma), and B.1.1.529 (Omicron) [15,17]. The L452R mutation appears in lineages B.1.617.2 (Delta), B.1.429 (Epsilon), and B.1.617.1 (Kappa) [18]. The K417N mutation appears in lineage B.1.351 (Beta), while the K417T mutation appears in lineages P.1 (Gamma) and B.1.1.529 (Omicron) [15,17]. The E484K mutation is present in lineages B.1.351 (Beta), P.1 (Gamma), P.2 (Zeta), B.1.525 (Eta), and B.1.526 (Iota), the E484Q mutation is present in lineage B.1.617.1 (Kappa), and the E484A mutation is present in lineage B.1.1.529 (Omicron) [15,19]. Therefore, it can be inferred that K417, L452, E484, and N501 in the RBD are key sites affecting the function of the S protein and are prone to mutation. In particular, three different mutations have appeared in the E484 site.

Previous studies have shown that different SARS-CoV-2 mutants have different mutations in the RBD of the S protein, causing changes in viral pathogenicity and infectivity [17,20]. This means that some vaccines currently in use may be less protective against some of the currently existing variants, which could lead to the widespread transmission of the mutated virus in the population, making it more difficult to control future COVID-19 outbreaks. Therefore, there is an urgent need to develop vaccines that confer strong broad-spectrum protection against existing or emerging mutated viruses. In this study, different mutant RBD proteins were expressed in an *Escherichia coli* expression system, and their antigenicity and immunogenicity were compared and evaluated in mice in order to screen candidates for vaccine design and drug targets and to facilitate virus antibody detection kit development.

## 2. Materials and Methods

### 2.1. Bacterial Strains, Construction, and Growth Conditions

*E. coli* XL1-Blue was used as a host to express the RBD protein. The bacterial strains in our experiment were cultured in LB medium at 37 °C. Ampicillin was added as needed. The fragment of the SARS-CoV-2 S protein gene (GenBank: NC_045512.2) (991–1749 bp) corresponding to amino acids 331−583 of the SARS-CoV-2 S protein (GenBank: YP_009724390.1) was synthesized. The expression vector pQE30 was digested with B*am*HI and H*ind*III restriction enzymes. The ClonExpress^®^ Ultra One Step Cloning Kit (Vazyme, Nanjing, China) was used to ligate the target fragment into the vector. Primers used for cloning and mutant construction are shown in Table 1.

### 2.2. Protein Expression and Purification

The recombinant strains were streaked on LB plates (containing 200 μg/mL ampicillin). Single colonies were inoculated in LB medium containing 100 μg/mL ampicillin and cultured overnight at 37 °C. Cultures were transferred to 1 L LB medium. When the OD600 value of the bacterial solution reached 0.5, IPTG was added at a final concentration of 0.5 mM and bacteria were cultured at 37 °C for 8–12 h. The cultured cells were harvested and resuspended in 15 mL lysis buffer (10 mM imidazole, 300 mM NaCl, 50 mM NaH_2_PO_4_, pH 8.0). Then, 0.5 mL lysozyme (50 mg/mL) was added and the lysate was placed on ice for 20 min. Ultrasonic disruption was performed with the following parameters: total time 16 min, working time 6 s, intermittent time 3 s, and power 300 W. After centrifugation for 20 min at 12,000 rpm and 4 °C, the supernatant was discarded, and the precipitate (inclusion body) was retained. Next, 20 mL of precipitation lysis buffer (8 M urea, 100 mM NaH_2_PO_4_, 100 mM Tris-HCl, pH 8.0) was added to the inclusion body. After sufficient oscillation, ultrasonication was carried out. The supernatant (containing protein after inclusion body dissolution) was collected by centrifugation for 20 min at 12,000 rpm and 4 °C, and the precipitate was discarded. The RBD protein was purified under denaturing conditions with HisSep Ni-NTA Agarose Resin. The inactive RBD protein was added to refolding buffer (100 mM Tris, 400 mM L-arginine, 2 mM EDTA, 5 mM GSH, 0.5 mM GSSG, 10% (*v*/*v*) glycerol, pH 8.0) and incubated for 12 h at 4 °C. Finally, the refolded RBD protein was concentrated and desalted by a 10 kDa ultrafiltration tube.

### 2.3. SDS-PAGE and Immunoblotting

The recombinant RBD protein was mixed with protein loading buffer, boiled for 5 min, and centrifuged for 10 min at 12,000 rpm. Next, proteins were separated by 12% SDS-PAGE (5 μL of supernatant per lane). For Coomassie Bright Blue staining, CBB Fast Staining Solution (Tiangen biotech, Beijing, China) was used. For Western blot (WB) analysis, the proteins were transferred from the gel to a nitrocellulose membrane by semi-dry membrane transfer (the membrane transfer parameters: 25 V, 1 A, and 25 min). The membranes were incubated in blocking buffer (5% BSA in TBST buffer (Tris 9.68 g/L, NaCl 32 g/L, 0.02% Tween-20)) at room temperature for 2 h. After washing the membrane with TBST buffer three times, the membrane was incubated with mouse monoclonal anti-His tag (1:5000, Clone: 9C11, Yeasen Biotechnology, Shanghai, China) or human monoclonal anti-RBD (1:1000, Clone: 7H6, KMD Bioscience, Tianjin, China) overnight at 4 °C. Following incubation, the membranes were washed and incubated with horseradish peroxidase (HRP)-conjugated goat anti-mouse (1:10,000) or HRP-conjugated goat anti-human for 1 h at room temperature. After washing, the membrane was immersed in a chromogenic solution and protein bands were imaged with a UVI gel imager (UVItec, Cambridge, UK).

### 2.4. Mouse Immunization

Ten RBD proteins were prepared and mixed with Freund’s adjuvants at a ratio of 1:1. In this experiment, three mice were immunized in each experimental group. Female BALB/c mice aged 6–8 weeks were subcutaneously immunized with 500 μL of RBD protein solution (30 μg of protein/mouse) or PBS and boosted on days 7 and 14. Blood was sampled on days 0 (prevaccination), 13, and 25. After coagulation at room temperature for 1 h, sera were collected by centrifugation (5000 rpm, 30 min, 4 °C) and stored at −80 °C until use.

### 2.5. Enzyme-Linked Immunosorbent Assay (ELISA)

ELISA was used to analyze antibody responses in serum. Microplate wells were coated with RBD protein (100 ng/well) in PBS coating buffer and incubated overnight at 4 °C. Plates were washed three times using PBST containing 0.1% Tween 20 and blotted dry. The plates were sealed at room temperature with 5% BSA for 2 h. Continuously diluted serum with PBS buffer was added to the microplate (100 μL/well), which was incubated for 1 h at 37 °C. Then, the plate was washed. HRP-conjugated goat anti-mouse (100 μL/well, 1:2000 in PBS) was added to each well, followed by incubation for 1 h at room temperature. Next, the plate was washed six times and incubated with 100 μL 3,3′,5,5′-tetramethylbenzidine (TMB) solution for 15 min. To stop the reaction, 50 μL of stop solution (1 M H_2_SO_4_) was added to each well, and Infinite M200 fluorescent multifunctional enzyme marker (Tecan, Männedorf, Switzerland) was used to read the absorbance at 450 nm.

### 2.6. Statistical Analysis

Data are expressed as mean ± standard deviation. Significant differences were determined by a one-way analysis of variance followed by the Tukey’s multiple comparison test. Data were analyzed and graphs were drawn using Origin software (version 8.0, OriginLab, Northampton, MA, USA) and GraphPad Prism software (version 5.0, GraphPad Software, San Diego, CA, USA). Each experiment was independently replicated three times and statistical significance was defined as *p* < 0.05.

## 3. Results

### 3.1. The Nine RBD Protein Mutants Were Obtained by Prokaryotic Expression and Affinity Chromatography

**Figure 1:**
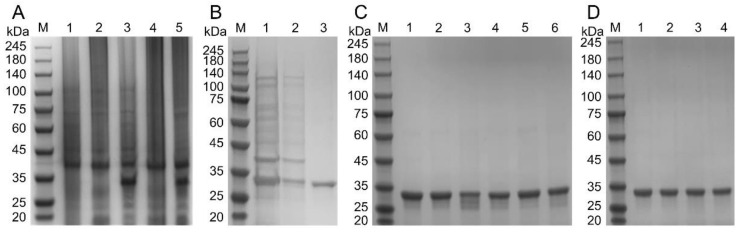
Expression and purification of the RBD protein mutants. (**A**) Lane 1: total protein of wild Enterobacter; Lane 2: total protein of uninduced recombinant strain; Lane 3: total protein of the recombinant strain after induction; Lane 4: supernatant protein after ultrasonic enucleation; Lane 5: precipitated protein after ultrasonic enucleation. (**B**) Lane 1: 8M urea dissolved total inclusion body protein; Lane 2: unbound protein collection solution; Lane 3: recombinant RBD protein collection solution. (**C**,**D**) SDS-PAGE analysis of denatured, renatured, and concentrated recombinant RBD protein and mutant protein. (**C**) Lane 1: mutant K417T; Lane 2: mutant K417N, Lane 3: mutant L452R; Lane 4: mutant E484K; Lane 5: mutant E484Q; Lane 6: mutant N501Y. (**D**) Lane 1: wild-type RBD protein; Lane 2: mutant L452R-E484Q; Lane 3: mutant K417T-E484K-N501Y; Lane 4: mutant K417N-E484K-N501Y.

The expression of the recombinant SARS-CoV-2 RBD protein was induced in *E. coli* XL1-Blue cells and detected by SDS-PAGE. Although the recombinant RBD protein could be expressed in *E. coli* cells, the protein existed in the form of an inclusion body (Figure 1A). Therefore, denaturation and refolding were required. A large number of induced bacterial cells were collected, and the inclusion body protein was obtained after cell rupture. After dissolution with 8 M urea, a Ni-NTA agarose flow column was used for purification (Figure 1B). When the purified denatured RBD protein was refolded, a recombinant RBD protein with biological activity was obtained, and the molecular weight of ≈30 kDa was consistent with the expected theoretical value. In this study, nine RBD protein mutants were constructed by reverse PCR [21], including six single-point mutants, K417T, K417N, L452R, E484K, E484Q, and N501Y; a two-locus mutation, L452R-E484Q; and two three-site mutants, K417T-E484K-N501Y and K417N-E484K-N501Y. In this experiment, affinity chromatography and ultrafiltration were used to remove the endotoxin in the recombinant protein. After repeated operations, the endotoxin content in all mutant protein solutions was less than 2.0 eu/mL. The SDS-PAGE results after denaturation, refolding, and concentration of the nine RBD protein mutants and wild-type are shown (Figure 1C,D).

### 3.2. The RBD Protein Mutants Possessed Antigenicity In Vitro

**Figure 2:**
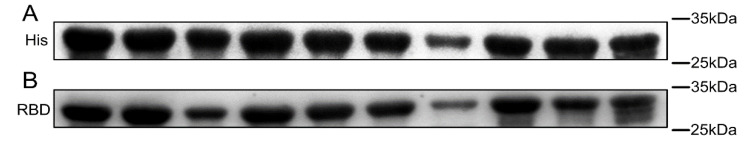
Western blotting analysis of RBD protein mutants. (**A**) His-tag mouse monoclonal antibody. (**B**) RBD human monoclonal antibody. The bands from left to right were wild RBD, K417T, K417N, L452R, E484K, E484Q, N501Y, L452R-E484Q, K417T-E484K-N501Y, and K417N-E484K-N501Y.

In order to ensure that the purified wild-type RBD protein and mutant proteins have a biological function, their specific antigenicity was analyzed using WB. Mouse monoclonal anti-His tag (Figure 2A) and human monoclonal anti-RBD (Figure 2B) were used as primary antibodies. The results show that a distinct single band is detected at approximately 30 kDa, which indicates that the wild RBD protein and nine purified mutant proteins had strong antigenicity and could be used as antigens for subsequent immune experiments.

### 3.3. The Mutants E484K, E484Q, K417T-E484K-N501Y, and K417N-E484K-N501Y Displayed Excellent Immunogenicity in Mice

**Figure 3:**
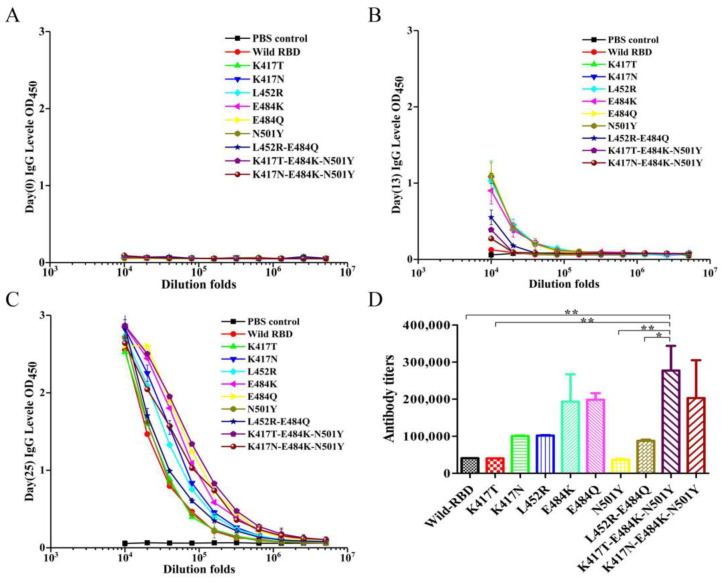
Anti-SARS-CoV-2 RBD antibody levels in mice by ELISA. It was coated with 100 ng of wild RBD protein, and after blocking with 2 times gradient diluted serum. (**A**–**C**) represent the antibody levels of RBD on days 0, 13, and 25, respectively. (**D**) The RBD antibody titers were expressed as the minimum concentration (maximum dilution) required for binding antigen. Statistical significance was defined as *p* < 0.05, and * *p* < 0.05, ** *p* < 0.01.

To evaluate the in vivo immunogenicity of the mutated RBD proteins, mice were immunized with both wild-type RBD and the nine RBD protein mutants, and blood samples were taken on days 0, 13, and 25. The total antibody titer against the SARS-CoV-2 RBD protein was evaluated by ELISA using HRP-conjugated goat anti-mouse as the secondary antibody. Before prevaccination, no anti-RBD was detected in the serum of all mice (Figure 3A). On day 13, anti-RBD was detected in mice immunized with both wild-type RBD and the nine RBD protein mutants (Figure 3B). On day 25, total antibody levels were significantly increased in all mice immunized with RBD and RBD mutant proteins (Figure 3C). After receiving three doses, the total resistance was greatly enhanced compared with the resistance levels after receiving only two doses. Interestingly, different RBD proteins yielded significantly different antibody titers after immunization (Figure 3D). On day 25, the antibody titers induced by mutants E484K, E484Q, K417T-E484K-N501Y, and K417N-E484K-N501Y were 4.7-, 4.8-, 6.8-, and 5.0-fold higher than those induced by wild-type RBD, respectively. These results indicate that the mutants E484K, E484Q, K417T-E484K-N501Y, and K417N-E484K-N501Y have excellent immunogenicity. In addition, the titers of mutants K417N, L452R, and L452R-E484Q were about 2-fold higher than that of wild-type RBD. However, surprisingly, the titers of mutants K417T and N501Y were approximately equal to that of wild-type RBD, suggesting that the single mutations at K417T and N501Y may not cause significant changes in the immunogenicity of RBD.

### 3.4. The Site E484 Has a Significant Impact on the Function of the RBD Protein

**Figure 4:**
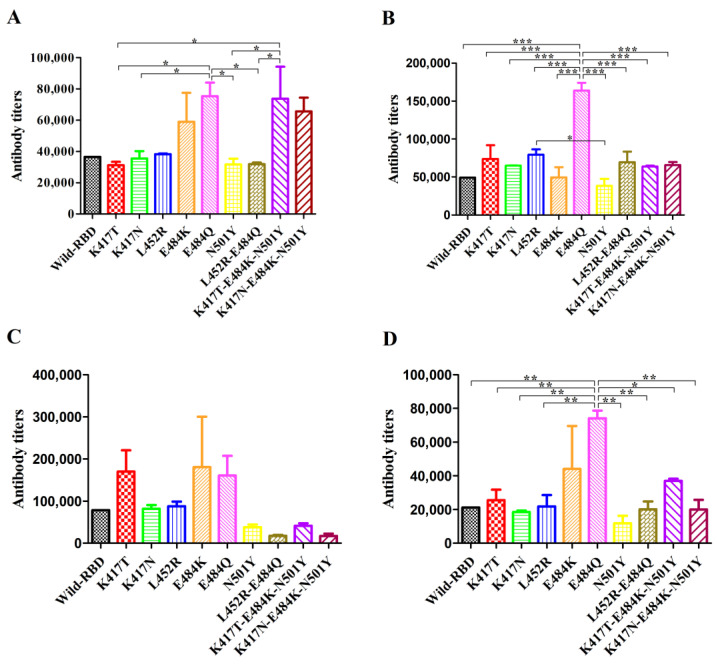
Extensive analysis of the antigenicity of RBD mutant protein. It was coated with 100 ng/well RBD mutant protein, and, after blocking, 2 times gradient diluted serum was added. (**A**) E484K protein. (**B**) E484Q protein. (**C**) K417T-E484K-N501Y protein. (**D**) K417N-E484K-N501Y protein. Statistical significance was defined as *p* < 0.05, and * *p* < 0.05, ** *p* < 0.01, *** *p* < 0.001.

To further evaluate the extensive antigenicity of mutants E484K, E484Q, K417T-E484K-N501Y, and K417N-E484K-N501Y, we coated microplate wells with E484K, E484Q, K417T-E484K-N501Y, and K417N-E484K-N501Y proteins, respectively, and after blocking, antibody titers in mouse serum collected on day 25 were determined. When RBD mutant proteins of E484K, E484Q, K417T-E484K-N501Y, and K417N-E484K-N501Y were coated separately as antigens, higher antibody titers were detected in the sera of mice immunized with E484Q (Figure 4B). These results indicate that the mutant E484Q can induce high levels of antibodies, and the antibodies induced by the mutant have high antigenic binding ability to a wide spectrum of mutant RBD proteins. After coating with E484K protein, a high antibody titer was detected in the sera of all immunized mice (Figure 4A), indicating that E484K as an antigen has a high binding ability to antibodies induced by wild-type and other mutant RBD proteins. Although antibody titers against RBD were detected in the serum of all immunized mice after coating with K417T-E484K-N501Y (Figure 4C) and K417N-E484K-N501Y (Figure 4D) proteins alone, there was no significant difference between the antibody titer produced after the mice were immunized with other mutant proteins, except for the mutants E484K and E484Q. This means that the mutant proteins K417T-E484K-N501Y and K417N-E484K-N501Y may not be suitable as antigens in antibody detection. In conclusion, the above results indicate that the mutant proteins E484K, E484Q, K417T-E484K-N501Y, and K417N-E484K-N501Y all have good immunogenicity and can induce antibody production more strongly than wild-type RBD protein in mice.

## 4. Discussion

The cumulative multiple mutations of SARS-CoV-2 have formed highly infectious Delta and Omicron variants [22,23], which will trigger another outbreak of the COVID-19 epidemic around the world, exacerbating the global pandemic and threatening public health. The SARS-CoV-2 Omicron variant is of particular concern because of its increased transmissivity and the high number of mutations in the spike protein, which have the potential to evade neutralizing antibodies induced by the currently used COVID-19 vaccines [24,25]. In addition, it has been shown that the mechanical stability of SARS-CoV-2 RBD protein was 250 pN, while that of SARS-CoV RBD protein was 200 pN, which may play an important role in increasing transmissivity of the COVID-19 pandemic [26].

The spike protein of SARS-CoV-2 consists of two major functional domains containing a total of 1273 amino acids. Located at the N-terminus of the S protein is the S1 functional region containing the NTD and RBD domains. The remaining part is the S2 functional region containing two trimeric structures mediating membrane fusion [4]. Therefore, the NTD and RBD domains in the S1 functional region are candidates for the development of vaccines or superior antigens [27,28]. Several SARS-CoV-2 mutants are currently in focus, such as the Alpha (B.1.1.7), Beta (B.1.351), Gamma (P.1), Delta (B.1.617.2), Kappa (B.1.617.1), and Omicron (B.1.1.529) variants. The binding affinity of their corresponding mutant RBD protein to ACE2 also changes. For example, the Alpha (B.1.1.7), Beta (B.1.351, Gamma (P.1), Kappa (B.1.617.1), and Omicron (B.1.1.529) RBD proteins showed a higher affinity for ACE2 than wild-type RBD. Alpha (B.1.1.7) and Kappa (B.1.617.1) RBD proteins had a much higher affinity for ACE2 than wild-type RBD, while the Delta (B.1.617.2) RBD protein had a lower affinity for ACE2 than wild-type RBD [17,22]. In addition, it is interesting that K417T-E484K-N501Y, K417N-E484K-N501Y, and K417T-E484A-N501Y mutant RBD proteins had a lower affinity for ACE2 than the N501Y mutant RBD [16,29]. E484 and N501 are key sites to improve the binding affinity between RBD and ACE2 [17,22]. However, Koelher et al. investigated some RBD mutations, which described the effect of receptor binding energetics and neutralization of the SARS-CoV-2 variants by atomic force microscopy and molecular dynamics. They found that N501Y and E484Q mutations were particularly important for greater stability of the RBD-ACE2 complex, but the N501Y mutations were unlikely to significantly affect antibody neutralization [30].

Several RBD mutants selected in this study were present in the above SARS-CoV-2 variants. The recombinant RBD protein was expressed by *E. coli*, and after purification by affinity chromatography, BALB/c mice were immunized and antibodies against RBD were detected in serum (Figure 3B,C). The results show that RBD protein expressed by a prokaryotic expression system had good immunogenicity although it could not be modified by folding and glycosylation. The mutants E484K, E484Q, K417T-E484K-N501Y, and K417T-E484K-N501Y all showed higher antibody titers than wild-type RBD (Figure 3D), indicating that these sites can significantly enhance the immunogenicity of RBD protein to induce the production of high levels of neutralizing antibodies. However, the mutant N501Y showed a lower antibody titer than the wild type (Figure 3D). Although many studies have shown that the mutant N501Y can significantly increase the binding affinity between RBD and ACE2 [17,22,29,31], its immunogenicity to RBD in our study was not enhanced but decreased. Therefore, we assume that the binding affinity between RBD and ACE2 may not be directly related to the immunogenicity of the RBD protein.

To verify the above hypothesis, we used four RBD mutants, E484K, E484Q, K417T-E484K-N501Y, and K417T-E484K-N501Y, as antigens and detected the antibodies against RBD in the serum of all immunized mice. The results show that single mutants E484K and E484Q, when used as antigens, could not only detect high antibody titers in the sera of all immunized mice but also detect higher antibody titers in the sera of mice immunized with mutants E484K and E484Q as compared with mice immunized with wild-type and other mutant RBD proteins (Figure 4). It has been shown that intratype antigenic variation due to mutation(s) is widely considered the main hurdle to appropriate FMD vaccine development, such that two substitutions of distantly located aa at B-C (T48I) and G-H (A143V) loops, in combination, distorted the VP1 G-H loop, which leads to the variation of the antigen [32]. The study of Huang et al. showed that single mutations L452R and F490S reduce the antigenicity to neutralizing antibodies [33]. The mutant E484Q can also significantly enhance the binding affinity between RBD and ACE2 [17,30], but the results of our study indicate that the mutant E484Q can also significantly enhance the immunogenicity of RBD protein. In addition, surprisingly, although the mutant E484K could not significantly enhance the binding affinity between RBD and ACE2, the results of this study show that the mutant E484K could also enhance the immunogenicity of RBD protein. The above results verify our conjecture that the binding affinity between RBD mutants and ACE2 does not determine the immunogenicity of the mutant as an antigen.

## 5. Conclusions

In a pandemic where a virus frequently mutates, the broad-spectrum effectiveness of neutralizing antibodies and vaccines is crucial, so these studies are now the focus of many researchers. In the present study, different RBD mutants were selected as immunogens to investigate the differences in the levels of antibodies induced by them, and two significant mutants, E484K and E484Q, were found. Therefore, it can be inferred that the E484 amino acid residue on RBD not only significantly affects the binding affinity with the receptor ACE2, but also has a significant impact on the immunogenicity of the RBD protein, and it may even affect the transmissibility and pathogenicity of the mutant virus.

## Figures and Tables

**Table 1:** List of primers used in this study.

Primers Name	Sequence (5′-3′)
RBD-F	TCGCATCACCATCACCATCACAATATTACAAACTTGTGCCCTTTTG
RBD-R	GAGTCCAAGCTCAGCTAATTTTACTCAAGTGTCTGTGGATCACGG
K417T-F	CAAACTGGAACCATTGCTGATTATAATTATAAATTACC
K417T-R	CAGCAATGGTTCCAGTTTGCCCTGGAGCGATTTGTC
K417N-F	CAAACTGGAAACATTGCTGATTATAATTATAAATTACC
K417N-R	CAGCAATGTTTCCAGTTTGCCCTGGAGCGATTTGTC
L452R-F	ATAATTACCGCTATAGATTGTTTAGGAAGTCTAATC
L452R-R	CAATCTATAGCGGTAATTATAATTACCACCAACCTTAG
E484K-F	AATGGTGTTAAGGGTTTTAATTGTTACTTTCCTTTAC
E484K-R	TTAAAACCCTTAACACCATTACAAGGTGTGCTACCG
E484Q-F	AATGGTGTTCAGGGTTTTAATTGTTACTTTCCTTTAC
E484Q-R	TTAAAACCCTGAACACCATTACAAGGTGTGCTACCG
N501Y-F	CCAACCCACTTACGGTGTTGGTTACCAACCATACAGAG
N501Y-R	CAACACCGTAAGTGGGTTGGAAACCATATGATTGT

## Data Availability

Not applicable.
